# Facile Fabrication of 3D Porous Sponges Coated with Synergistic Carbon Black/Multiwalled Carbon Nanotubes for Tactile Sensing Applications

**DOI:** 10.3390/nano10101941

**Published:** 2020-09-29

**Authors:** Yousef Al-Handarish, Olatunji Mumini Omisore, Wenke Duan, Jing Chen, Luo Zebang, Toluwanimi Oluwadara Akinyemi, Wenjing Du, Hui Li, Lei Wang

**Affiliations:** 1 Research Centre for Medical Robotics and Minimally Invasive Surgical Devices, Shenzhen Institutes of Advanced Technology, Chinese Academy of Sciences, Shenzhen 518055, China; omisore@siat.ac.cn (O.M.O.); wk.duan@siat.ac.cn (W.D.); jing.chen@siat.ac.cn (J.C.); zb.luo@siat.ac.cn (L.Z.); tolu@siat.ac.cn (T.O.A.); wj.du@siat.ac.cn (W.D.); hui.li1@siat.ac.cn (H.L.); 2 Shenzhen College of Advanced Technology, University of Chinese Academy of Sciences, Shenzhen 518055, China; 3 Shenzhen Institutes of Advanced Technology, University of Chinese Academy of Sciences, Shenzhen 518055, China; 4 CAS Key Laboratory for Health Informatics, Shenzhen Institutes of Advanced Technology, Chinese Academy of Sciences, Shenzhen 518055, China

**Keywords:** flexible tactile sensors, piezoresistive sensors, 3D porous structure, healthcare systems, human–machine interface

## Abstract

Recently, flexible tactile sensors based on three-dimensional (3D) porous conductive composites, endowed with high sensitivity, a wide sensing range, fast response, and the capability to detect low pressures, have aroused considerable attention. These sensors have been employed in different practical domain areas such as artificial skin, healthcare systems, and human–machine interaction. In this study, a facile, cost-efficient method is proposed for fabricating a highly sensitive piezoresistive tactile sensor based on a 3D porous dielectric layer. The proposed sensor is designed with a simple dip-coating homogeneous synergetic conductive network of carbon black (CB) and multi-walled carbon nanotube (MWCNTs) composite on polydimethysiloxane (PDMS) sponge skeletons. The unique combination of a 3D porous structure, with hybrid conductive networks of CB/MWCNTs displayed a superior elasticity, with outstanding electrical characterization under external compression. The piezoresistive tactile sensor exhibited a high sensitivity of (15 kPa^−1^), with a rapid response time (100 ms), the capability of detecting both large and small compressive strains, as well as excellent mechanical deformability and stability over 1000 cycles. Benefiting from a long-term stability, fast response, and low-detection limit, the piezoresistive sensor was successfully utilized in monitoring human physiological signals, including finger heart rate, pulses, knee bending, respiration, and finger grabbing motions during the process of picking up an object. Furthermore, a comprehensive performance of the sensor was carried out, and the sensor’s design fulfilled vital evaluation metrics, such as low-cost and simplicity in the fabrication process. Thus, 3D porous-based piezoresistive tactile sensors could rapidly promote the development of high-performance flexible sensors, and make them very attractive for an enormous range of potential applications in healthcare devices, wearable electronics, and intelligent robotic systems.

## 1. Background Study

In the recent years, advances in artificial intelligence and the internet of things have made it obvious that high performance flexible tactile sensors are a crucial sensing element, and they have become a research hotspot with growing demands in the electronic industry, with enormous practical applications, including personalized health-care monitoring systems [1,2,3,4], electronic skin [5,6,7], and human–machine interactions [8,9,10,11]. Primarily, tactile sensors are applied in robotic systems to imitate human perception systems of a diverse range of external pressures and to perform interventional tasks. Such tasks are grasping, detecting object shapes, and sensing a wide pressure range including the low-pressure region (>10 kPa), medium-pressure region (10–100 kPa), and even high-pressure region (>100 kPa) [12,13].

The main criteria to evaluate tactile sensors performance are, but not limited to, high sensitivity, fast response time, wide detection range, and excellent stability. In general, conventional tactile sensors based on semiconductors [14] and metals foils [15] have shown drawbacks in terms of sensing limited range and complexity of fabrication process. These demonstrate the lack of functionality of the sensing materials, which limit their practical applications [16]. Recently, a variety of flexible tactile sensors have been developed based on different operational sensing mechanisms, including piezoresistive-based [17,18], piezoelectric-based [19,20], capacitive based [21,22], and optical based sensors [23]. Among them, flexible piezoresistive sensors, which are based on transducing the external mechanical pressure into resistance variation outputs, have attracted considerable attention, due to their simple structure, low-cost, and easy signal processing [9,24]. Although, significant progress has been achieved in manufacturing tactile sensors with high performance, it is still challenging to obtain a sensor capable of quantifying and measuring a wide pressure range, including tiny stimulus, with high sensitivity.

In order to improve the sensitivity of piezoresistive tactile sensors, some research studies have been performed, and results from several works have demonstrated various fabrication strategies, rather than the conventional method of the micro-pyramid array structure [25,26]. These include use of 3D printing [27], micro-pillar structure [17], chemical vapor deposition [28], sponge structure [20,29], and the dip-coating process [30,31]. Amongst these design strategies, the dip-coating of the conductive nanoparticles on the surface of polymer-based substrate has been in growing demand for fabricating flexible piezoresistive sensors. Previous studies showed that this strategy can be achieved through a scalable, low-cost processing technology [25,32,33,34]. In addition, commercial sponges proved to be ideal candidates for the three-dimensional (3D) porous structure used in preparing high-performance piezoresistive sensors via the dip-coating process [35,36]; while polydimethylsiloxane (PDMS), as a thermosetting polymer with good flexibility and elasticity, has been in demand for improving the compressibility of pressure sensors. In addition, a variety of nanoconductive composites such as carbon black (CB), carbon nanofibers (CNFs), graphene, carbon nanotubes (CNTs), and silver nanowires (AgNWs), have been proved, and widely used, as optional sensing for piezoresistive sensors [37,38,39]. For instance, CB and CNTs attracted much attention due to their good conductivity, large aspect ratio, and with excellent mechanical properties under repeated external pressure. These sensors can play an important role in increasing the interfacial area between the conductive fillers and polymer matrix [40,41].

Moreover, the novelty of 3D porous conductive structures increases the infiltration of conductive fillers into skeleton walls, and decreases the distance between cells upon external deformation or compressive strain. This normally results in variation of relative resistance [42,43]. The excellent viscoelastic features and the good porosity of the 3D porous conductive composites generate significantly stable resistance signals over a wide pressure range [44,45]. An example is the combination of a polymer matrix of 3D porous PDMS foam with a synergetic conductive network, which became an innovative facile technique for fabricating the high-performance piezoresistive sensors [46]. The literature has indicated the importance of the synergetic effect of the hybrid conductive fillers to generate significant resistance variation over a wide range. The piezoresistive sensing principle is mainly based on the deformation of the electrically conductive polymer nanocomposite under an applied external pressure, causing the variation of electrical resistance. Thus, combination of CB and multiwalled carbon nanotube (MWCNT) nanofillers has an excellent advantage, including enhancing their sensitivity and detectability with rapid-response, even in a small strain region. For example, Xu et al. reported a new facile method to prepare a stretchable and conductive 3D porous PDMS/CNF nanocomposite by immersing CNF-coated sugar particles into PDMS substrate. The as-prepared piezoresistive pressure sensor exhibited excellent sensitivity and durability over an increased detection range of up to ~94% compression strain [47]. Furthermore, Zhai et al. reported a facile and cost-effective strategy to prepare a 3D porous sensor based on CB/PDMS. The sensor exhibited high sensibility, excellent stability, and a large compression range of up to 91% [48]. Similarly, Ma et al. successfully used MWCNT/RGO synergistic conductive networks with polyurethane sponges to fabricate lightweight porous piezoresistive sensors, with high sensitivity, outstanding compressibility (up to 75%), and very low densities (0.027–0.064 g cm^−3^) [20]. Recently, Zhao et al. presented a robust highly sensitive pressure sensor bioinspired by the *Epipremnum aureum* leaf and using sugar as a porous template. The reported pressure sensor exhibited a high sensitivity of 83.9 kPa^−1^ with a low detection limit of 0.5 Pa and excellent long-term durability (>28,000 cycles) [49].

Therefore, the high-sensitivity flexible piezoresistive tactile sensor with excellent working stability, rapid response, and low-detection limit is still widely desirable. Herein, a rapid response, ultra-wide detection range, and highly sensitive piezoresistive tactile sensor was integrated, based on a 3D porous dielectric layer, using a facile and cost-efficient strategy. The microporous dielectric layer was fabricated by a simple dip-coating CB/multiwalled carbon nanotube (MWCNT) synergetic network on porous PDMS sponge via ultrasonication. The synergetic conductive nanoparticles of CB/MWCNTs were dispersed both inside and outside the pore channels, to significantly enhance the sensing performance, electrical performance, and the stability of the 3D porous dielectric layer upon external compression. A scanning electron microscope (SEM) was employed to characterize the dielectric layers of the microstructure of the CB/MWCNTs/PDMS dielectric layer, and the sensor’s dynamic durability was also studied. In addition, the as-prepared piezoresistive tactile sensor, developed with the proposed strategy, was tested under various compressive strains to investigate its sensing performance and detecting range. Benefiting from superior performance, the piezoresistive tactile sensor was applied in monitoring various human biological signals including heart rate pulses, wrist bending, knee bending, and respiration. Additionally, finger grabbing motion was demonstrated to illustrate its prospective applications in human–robotic interface.

## 2. Experimental Section

Details of the proposed design and fabrication strategy, including the procedural steps applied, are discussed in this section. 

### 2.1. Materials

We utilized the following materials for the fabrication process of our proposed sensor. Carbon black (CB) of the model XF115 was purchased from XFNANO, Nanjing China O: XF115. The suspension of the CB is 30 × 45 nm, and the mass density 280 × 300 g/L. The carboxy-functionalized multi-walled carbon nanotubes (MWCNTs) powder was purchased from Chengdu Organic Chemicals Co., Ltd. (No.16, South section 2, the first Circle road, Chengdu, China). The commercial sugar cubes with size 18 × 18 × 10 mm^3^ were purchased from a local supermarket, and were employed as templates. The base agent and curing agent (Sylgard 184 silicone elastomer) of PDMS were obtained from Dow Corning Co., Ltd. (Unit B, 8/F, Golden Tower, 258 Tongren Road, Shanghai, China). Isopropanol alcohol (IPA) was purchased from Sigma-Aldrich (Unit B, 8/F, Golden Tower, 258 Tongren Road, Shanghai, China). All materials were used as received from the suppliers without any further purification.

### 2.2. Preparation of Porous Sugar/PDMS Sponge

**Figure 1:**
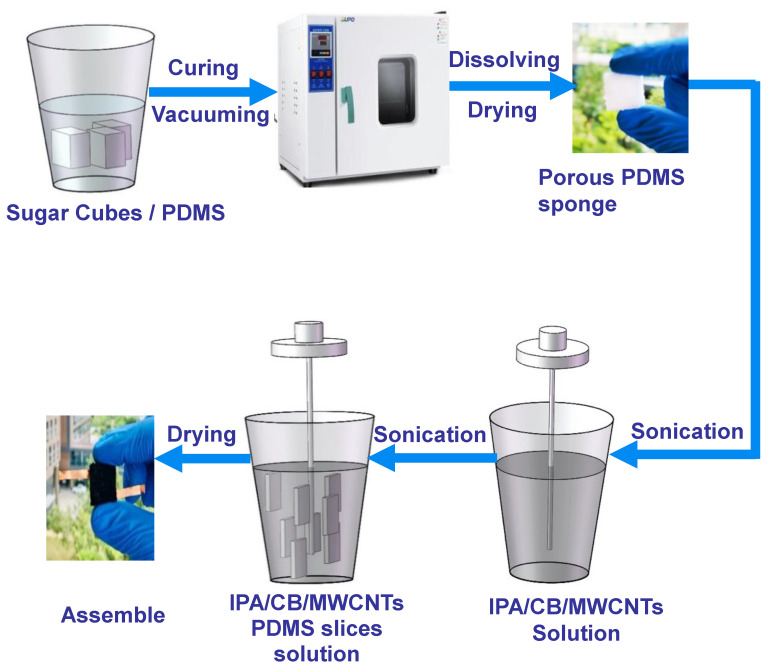
Schematic diagram of the fabrication process of the flexible piezoresistive tactile sensor based on 3D microstructure with carbon black (CB)/multiwalled carbon nanotube (MWCNT) conductive network. PDMS = polydimethysiloxane.

The fabrication approach of preparing a flexible tactile sensor with a 3D porous CB/MWCNTs/PDMS microstructure sponge is schematically shown in Figure 1. The adopted simple fabrication strategy, low-cost, and outstanding performance could be desirable in future for reliable tactile sensor fabrication. The process involves using commercially available sugar cubes as a sacrificial template. First, the PDMS mixture was formulated by mixing a curing agent with the base elastomer at a weight ratio of 1:10. This PDMS precursor was magnetically stirred for 30 s, after which it was degassed in a vacuum oven for 30 min to remove any unwanted bubbles. Next, the sugar cubes were immersed in a degassed PDMS precursor to facilitate the infiltration, and the mixture was dipped in a vacuum desiccator for 2 h. Then, the PDMS loaded sugar cubes were cured in a convection oven at 80 °C for 2 h. Next, the sugar cubes were trimmed away from the cured PDMS, and the PDMS-loaded sugar cubes were cut into the desired sizes (8 mm in width, 8 mm in length, and 2 mm in thickness) by using a razor blade. In order to dissolve sugar particles and obtain the 3D porous structure, the PDMS-loaded sugar slices were dipped in warm deionized water and stored in the oven at 60 °C for 30 min, and then released to dry at room temperature. Finally, the 3D PDMS porous structure was obtained.

### 2.3. Preparation of CB/MWCNT Composite Solution

In order to deposit the conductive layer on the 3D porous PMDS sponge, CB and MWCNT conductive fillers in a 1:1 weight ratio (0.225 g:0.225 g) were sonicated in 80 mL of isopropanol alcohol suspension, sequentially, after 1 h of sonication was carried out to obtain homogeneous dispersion of the CB/MWCNTs.

### 2.4. Fabrication of the 3D Porous Tactile Sensor

The dried 3D porous PDMS slices were treated by oxygen plasma with 100 W and power for 30 s to increase the infiltration of the conductive nanocomposites. Then, the 3D porous PDMS slices were immersed in the conductive solution and sonicated for 1 h, followed by drying in an oven for 1 h at 60 °C. The 3D porous CB/MWCNTs/PDMS nanocomposites were obtained and prepared for implementation in a high performance piezoresistive tactile sensor. Then, copper wires were bonded onto the two opposite sides, using commercial polyimide tape (PI). Finally, the device was sandwiched between two covering layers of PI for further studying of the electrical and mechanical characteristics.

### 2.5. Device Characterization

The morphology and microstructure of the 3D porous CB/MWCNTs/PDMS were characterized by carrying out scanning electron microscopy (SEM, Phenom XL). The scanning was operated at 5 kV to observe the surface and internal structure of the composite. This was done by cutting a layer of sensor sample into small slice (3 mm × 3 mm × 2 mm), and each slice was processed by spraying gold onto it, and then placed in the proper place for characterizing in situ morphology. Electrical resistance measurements were carried out using an automated source meter (Keithely 4200) for recording resistance change and real time continuous measurement of loading/unloading pressure sensing. The mechanical properties and cyclic stability were measured by applying an external compression using a Mark-10 combined with the source meter (Keithely 4200). Testing response of the designed sensor to applied pressure ranges was performed by controlled experiments. For this, different pressure values were gently loaded, and the resulting data sensed were acquired for comparison. Further investigations were also carried out to demonstrate the capability of the piezoresistive tactile sensor for human vital physiological signals, and in human–robot tactile sensing and feedback. For these, samples of the sensors were mounted to a wrist, finger, knee, and index fingertip to recognize and measure arterial pulses, wrist activities, and tactile pressure data, with fast response, to the touch of different objects.

## 3. Results and Discussion

### 3.1. Morphological Characterization

**Figure 2:**
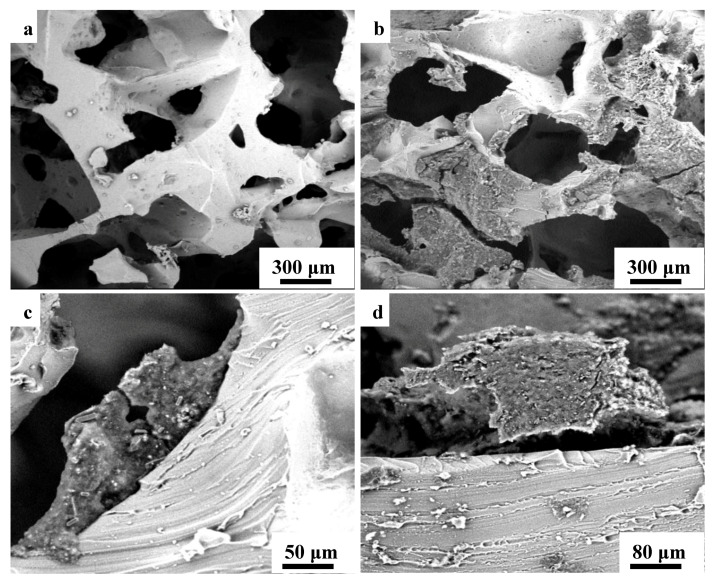
(**a**) SEM image of the pure PDMS sponge. (**b**) Morphological image of PDMS foam coated by CB/MWCNTs conductive composite. (**c**,**d**) Coated PDMS at different magnifications with a cross-sectional skeleton, and surface of pore walls covered by CB/MWCNTs conductive composite.

The scanning electron microscopy (SEM) images of the microstructure porous PDMS/MWCNTs/CB composites are shown in Figure 2. The morphological evolution was observed for the 3D structure, and it was found that it exhibited an average pore diameter between 148 and 286 μm, and with a mean average width skeleton between 32 and 58 μm. Figure 2a,b, show the SEM micrograph of the PDMS sponge before and after dip-coating by the conductive nanocomposite CB/MWCNTs, respectively. It can be clearly seen that the PDMS sponge obtains a smooth surface, with interconnected open-cells which construct an informal 3D porous structure. As can be confirmed in the corresponding magnified images shown in Figure 2c,d, the conductive nanoparticles attached themselves onto the rubber skeleton. At the same time, the well dispersed conductive nanocomposite CB/MWCNTs solution homogeneously stacked through the inner cell walls of the 3D porous structure, and uniformly wrapped on the skeleton surface. This makes the sensor’s surface, which becomes rough after sufficient ultrasonication treatment. Thus, owning to the CB/MWCNT nanoparticles and the obtained roughened surface, great electrical and mechanical properties can be expected. The synergetic effect of the combination of MWCNTs and CB nanoparticles enabled uniform dispersion of both carboxy groups between each other, which resulted in preventing the formation of large-size conductive fillers that could close the pores cells. By further magnifying the pore surface, it can be seen that the synergetic effect of the different dimensional conductive fillers forms a network structure, and improves its infiltration into the 3D skeleton. The pores were conformally coated with conductive fillers, and the empty inner cells remained open in appearance and in contact with each other under loading, to generate more conductive channels, resulting in the improvement of the conductivity and a high sensitivity.

### 3.2. Basic Working Principle and Sensing Performance

**Figure 3:**
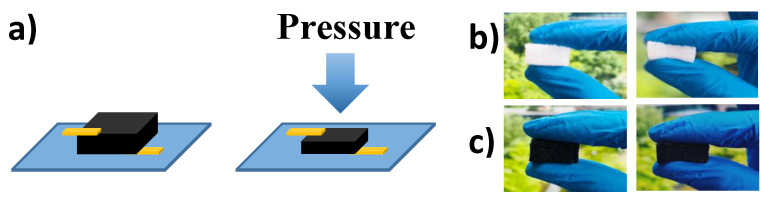
(**a**) Working principle schematic of the 3D porous piezoresistive sensor under external pressure; (**b**) photographs of the 3D porous structure layer of the pure PDMS sponge under relaxation (left) and compression (right); (**c**) photographs of the 3D porous dielectric layer of CB/MWCNTs/PDMS composites under relaxation (left) and compression (right).

Working schematics of the proposed piezoresistive tactile sensor based on a 3D porous CB/MWCNTs/PDMS dielectric layer are illustrated in Figure 3. For its sensing mechanism, the 3D porous structure consists of micro open cells with a smooth inner surface, which can be coated by conductive nanoparticles of CB/MWCNTs, to form a conductive rough surface and conductive inner channels, owing to their exceptional electrical properties. In order to investigate how the thickness of the dielectric layer and the microporous structure affect the sensing performance, a normal force was applied to the rough surface of the piezoresistive sensor. Figure 3b shows how the whole contact surface was deformed and pressed under external applied force. Once the sensor is under contact with a large force, the internal distance between the inner pores close, and conductive particles attach, and this results in decreasing the resistance. By removing the external force, the 3D skeleton recovers and obtains the original structure which enables nanocomposite cells to reach their initial resistance. The high sensitivity of the 3D porous tactile sensor was understood as the applied force, or increased contact area, leading to an increased variation in the sensor’s resistance, owing to decreased distance between the conductive particles in the CB and MWCNTs. Generally, most of the nonconductive particles are located in the inner surface of the pore channels. Hence, any change in the overlapping area between conductive materials results in a rapid change in the overall resistance.

The main factors considered to evaluate the performance of the proposed 3D porous piezoresistive tactile sensor are the sensitivity, rapid response time, and durability. Therefore, performance of the sensor can be described according to the value of sensitivity of piezoresistive cells (S), which is quantitatively defined as the ratio of the curves:

(1) S=δ(ΔI/I0)δP=δ((UR1−U/R0)/(U/R0))δP

We can further simplify the above equation to

$$S=\;\frac{\partial \left(\Delta I/I_{0}\right)}{\delta P}=\;\frac{\delta (-\Delta R/\left(\Delta R+R_{0}\right)}{\delta P}$$

where *U* is voltage, being a constant value, and *R*_1_ is the real-time resistance with the applied force. Additionally, *I*_0_ and *R*_0_ are the initial current and resistance, respectively, Δ*I* and Δ*R* are the change in measured current, and resistance with the applied force, and *P* is the applied pressure to the sensor. According to Equation (1), the sensitivity of the proposed tactile sensor can be derived from the analysis of experimental data. The calculated results can be divided into two regions, depending on the applied pressure. The sensitivity reached 15 kPa^−1^ in the low-pressure range of <10 Pa, while in the high-pressure range of <200 Pa, the sensitivity decreased from 15 kPa^−1^ to 10.5 kPa^−1^. The lower sensitivity can be ascribed to both the increased contact area between the flexible electrodes and the nanomaterials, and the increase of synergistic effect. Thus, the sensor sensitivity can be adjusted by regulating the contact area, the porous structure, and the ratio of nanocomposites in the 3D sensor.

### 3.3. Electrical and Piezoresistive Characterization

**Figure 4:**
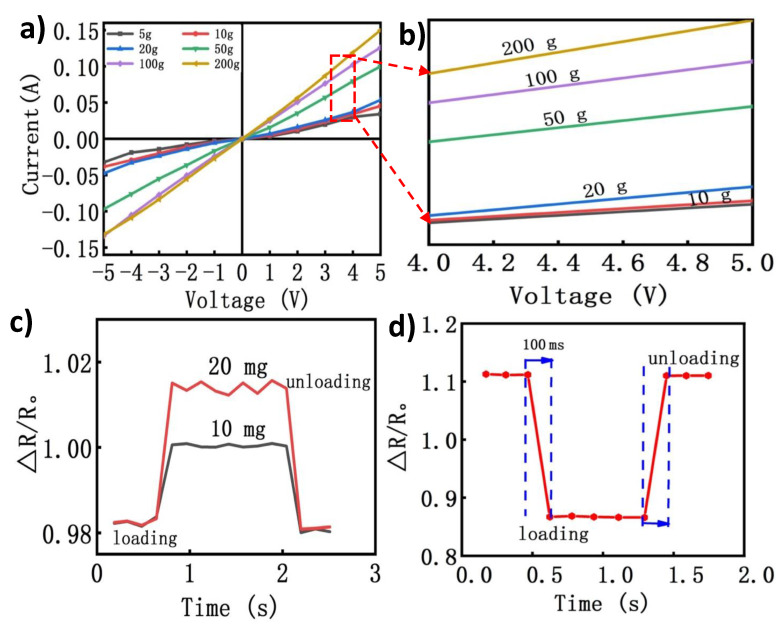
(**a**) Current–voltage curves (I-V) of the 3D porous conductive composite over the range from −5 to 5 V under different strains; (**b**) a magnified view of (**a**); (**c**) detection the response to micro-pressure of 10 mg and 20 mg; (**d**) real-response time of the 3D porous piezoresistive tactile sensor, with an applied pressure of 5 Pa under a loading and unloading process.

In order to further investigate the sensing performance of the proposed sensor, a series of different tests were conducted to analyze the key characteristics, effective requirements, and usefulness of the piezoresistive tactile sensor. The electrical property was assessed under different normal pressures. All samples were sandwiched between adhesive polyimide tape film to obtain stable signals, and the two copper wires were connected to the source voltage meter so as to form a circuit. The signal outputs were collected and displayed by using a digital multimeter (Kethley 4200). Figure 4a, illustrates the current–voltage curves of the 3D porous CB/MWCNTs/PDMS nanocomposites under different mass loads. For testing the current–voltage characteristics, the voltage was recorded in a range of −5 to 5 V, with an interval of 1 V. As shown in Figure 4a,b, the resistance gradually decreased, with the pressure increasing in a pressure range of 0–200 g. Clearly, the measured current–voltage curves exhibited a good linearity, when the voltage increased from −5 V to 5 V. This indicates that the CB/MWCNTs/PDMS pressure sensor has an outstanding reliability, which can make it suitable for numerous applications. Correspondingly, with increasing the applied voltage and loaded mass, the current–voltage curves exhibited a typical ohm’s law characteristic and linear relationship, owing to the decrease in electrical resistance, which depends on the applied pressure. Low detection limit and response time are also important parameters for the piezoresistive tactile sensor. The capability for detecting tiny stimuli was measured by slightly applying lightweight masses as static pressures of 10 mg and 20 mg, respectively. As shown in Figure 4c, it can be clearly seen that the device was able detect a very small stimulus and translate it into readable signals, with regular resistance curves during the loading and unloading process. Additionally, the relative resistance change of the flexible tactile sensor also exhibited a rapid dynamic response and recovery time. The real-time relative resistance variation curve of the device was measured by loading and unloading external pressure.

As shown in Figure 4d, the acquired resistance variation was achieved rapidly, with a response time of 100 ms under loading pressure of 10 Pa. Similarly, when the pressure was unloaded the resistance outputs could recover to the initial level within the same time of 100 ms. However, the actual response and recovery time could be faster by regulating the device setting of holding and releasing speeds. The rapid response time of the developed sensor was mainly exhibited owing to the unique elastic porous structure, where the relative resistance decreased under the loading due to the decrease in distance between the pore walls and the increase in the contact of conductive nanocomposites. Subsequently, the 3D porous skeleton gradually recovers due to the separation of the contact cell walls after releasing the applied pressure. Therefore, the unique 3D structure of the piezoresistive tactile sensor with inner empty pores can maintain an excellent elasticity and flexibility with a short response time. This confirms that the sensor could effectively detect a wide range of mechanical stimuli, with negligible viscoelastic behavior, which holds promise for its positional applications in detecting low pressure signals.

### 3.4. Strain Characterization

**Figure 5:**
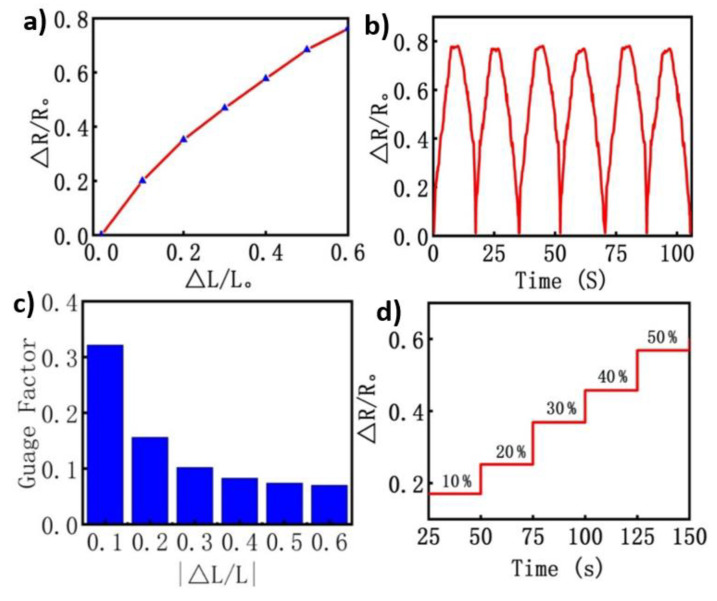
The typical mechanical sensing performance: (**a**) relative resistance change and the applied compressive strain as function of pressure at the rate of 5 mm/min; (**b**) plot showing stability of the normalized resistance change under repeatedly applied strain; (**c**) bar chart plotted for the calculated GF of the 3D porous piezoresistive tactile sensor, conductive for the various applied compressive strains (10% to 60%); (**d**) response of the sensor when the applied strain was gradually increased up to 60%.

Besides electrical characterization, the mechanical properties of the piezoresistive tactile sensor were also studied under compression loading conditions to investigate its strain capability for different potential applications. To assess the mechanical behavior of CB/MWCNTs/PDMS foam, a series of compressive cyclic loading tests were applied at different cyclic loading strains, up to 60%. A step strain (*ε* = 10–60%) was applied at a constant frequency of 5 Hz, and correspondingly the electrical response (Δ*R*/*R*_0_) decreased with an increase in the applied strain and with return to the initial level after removing the mechanical load. Initially, a pre-compression of 1% was applied in order to avoid the initial problems such as sliding/or settling of the conductive network [50]. As shown in Figure 5a, there can be clearly seen, a linear elastic relationship between the electrical response value (*ΔR*/*R*_0_) with the increase of the applied strain. The CB/MWCNTs/PDMS responded quickly and linearly at the first strain region, and with the increased strain gradually changed. At the same time, it obtained a stable recovery behavior, to its initial pre-compression position, immediately after the mechanical load was released, owning to the porous structure and low density, excellent recoverability, and compressibility, with outstanding structural performance, as demonstrated in Figure 5b. Gauge factor (GF), defined as the a ratio of the nanocomposite foam to the compression strain, is a critical factor used to evaluate the sensitivity of the sensor. It can be calculated as [45,51,52]:

(2) $$GF=\frac{\Delta R/R_{0}\;}{\begin{array}{c} \varepsilon \end{array}}$$

where, *ε* denotes the applied strain, and *R*_0_ and Δ*R* refer to the initial resistance, and the resistance change towards the real-time strain, respectively. Assessing the strain performance using Equation (2), the CB/MWCNTs/PDMS foam piezoresistive sensor showed a maximum value of GF = 0.32 when the cyclic strain was 10%, and when increasing the strain up to 60%, the GF decreased to 0.07.

The GF for each compressive strain was calculated and all GF values are plotted in one bar graph in Figure 5c. The gauge factor values can be identified as a linear response to the increased compression strain. The evaluation of gauge factor for the developed CB/MWCNTs/PDMS foam showed that the GF would increase accordingly with the applied strain. This could be attributed to the continuous press on the sensor which impacted the conductivity pathway due to the decreasing distance between nanoparticles. In addition, the resistance curve increased step by step with higher compressive strain levels, which is reflected by the decreased gauge values in Figure 5d. Similarly, the developed sensor exhibited a superior sensitivity, which is likely due to the microstructure of the piezoresistive sensor, which plays a critical role in the sensor’s performance. Again, strain was applied repeatedly to the sensor in a 10% iterative, step-wise (*ε* = 10% up to 60%), whereas the outputs were recorded for at least 30 cycles and analyzed offline. The porous sensor generated a stable resistance value for a different wide range of the applied strain values, and an outshoot was observed during the loading stage. To conclude this evaluation process, the working stability and reliability were characterized as the strain values by observing the electrical response to the repeatedly compressed strain. Furthermore, a slight overshoot was observed when the machine returned to its in initial position (*ε* = 0%). The peak conditions observed can be associated to the robust structure of the sensor and this triggers contacts between the foam sensor and the conductive electrodes, and the sensor recovered to its original state. Generally, the piezoresistive sensor showed an outstandingly stable electrical output signal under different deformations for long periods of time, and with anti-fatigue ability.

### 3.5. Device Reliability and Stability

**Figure 6:**
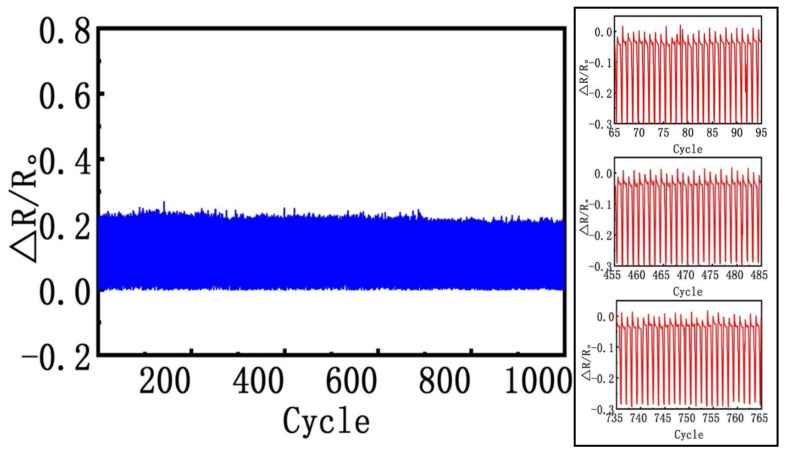
Durability and long-term stability of the 3D porous piezoresistive tactile sensor; The relative resistance change over 1000 cycles under continuously loading-unloading cycling and several magnified waveforms.

One of the main parameters to evaluate the performance of piezoresistive tactile sensors is outstanding reliability and stability under long-term loading/unloading conditions. To investigate that the proposed sensor is capable of these features, we further investigated the stability of the sensors under a repeated series of dynamically loading and unloading experiments. The sensor was bonded to a polyimide (PI) film tape in order to have the pressure applied from the rear side of the polyimide, and to protect the sensor from crashing. It was found that the sample immediately recovered to its original shape after releasing the pressure. As shown in Figure 6, the piezoresistive tactile sensor maintained a favorable stability and durability under pressure, even up to 1000 loading and unloading pressure, without any noticeable changes in the performance. Additionally, we observed the structure by magnifying the sensor’s images after the loading and unloading process. It was found from the magnified images, that the sensor maintained stable cycles along the testing period for 18 h while repeatedly loading and releasing pressure. Thus, the sensor could be an appropriate candidate for long-term practical applications. Furthermore, the piezoresistive tactile sensor showed a reasonably consistent response in its electrical resistance under the compressive cyclic force loads. Again, we found that the sensor structure remained intact and functional, which indicates that the developed sensor has a long lifetime and reliability. The performance’s key parameters, described in this work, were used to compare our proposed sensor against some recently reported piezoresistive sensors, as summarized in Table 1.

### 3.6. Applications in Human Physiological Detection

**Figure 7:**
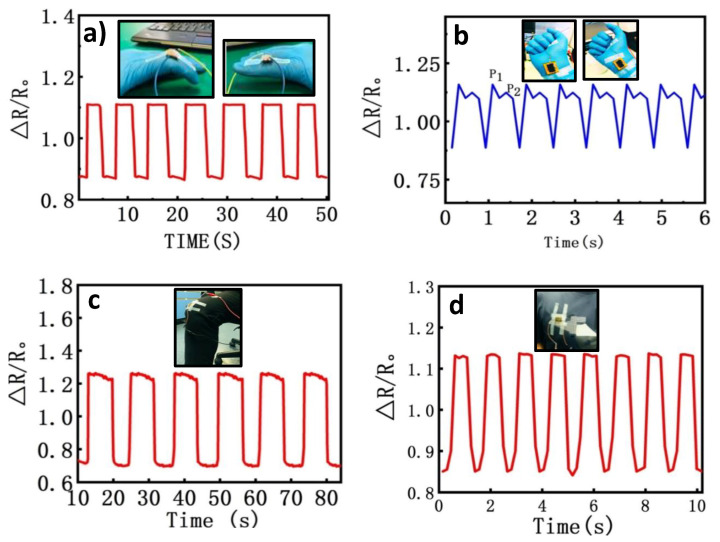
Schematic diagram of the piezoresistive tactile sensor in detecting different human physical signals. (**a**) Heart-rate pulses by attaching the sensor to a finger; (**b**) detection wrist pluses; (**c**) knee bending; (**d**) detection of the respiration rate.

Applicability of the developed 3D porous structure piezoresistive tactile sensor was investigated for sensing the broad pressure ranges that are exhibited in different daily activities, from personalized usage, to industrial and medical integration. In this study, we deemed that the verified high sensitivity and elasticity performance of the sensor holds great promise for the wearable application domain. Aside from the recent perspective of tactile sensors for interactive human–machine interfacing, flexible piezoresistive sensors also hold great promise for monitoring human physiological signals such as wrist pulse, motion movement, and breathing. To demonstrate the remarkable sensing capabilities of our proposed piezoresistive tactile sensor, customized studies were carried out for detection of a few physiological signals, using the experimental setup in Figure 7. During these studies, the proposed sensor was applied to different parts of several subjects to detect various human physiological signals, and the electrical responses were recorded.

First, the developed sensor was fixed at the index finger joint with a surgical tape to monitor the forefinger movement, as shown in Figure 7a. The flexible sensor could bend along with the finger movements, and the change in its relative resistance was recorded in real-time as the finger was periodically bent to reach angles of 45° and 90°, and further released to the initial state which is an angle of 180°. The corresponding relative resistance change provided a good recognizability of the bending and unbending status of the finger. Thus, the results obtained (Figure 7a) show that the sensor could detect the finger’s real-time response to different stimuli causing movements at different angles. Hence, we interpreted that the sensor is a good candidate for wearable devices. In a second case, the piezoresistive tactile sensor was applied for heart monitoring, by evaluating human heart rates. The common, conventional way is measuring the wrist pulse rate to estimate the physical condition of the human body. Thus, our flexible sensor was mounted around the radial artery of the subject’s wrist using medical adhesive tape, and the pulse rhythms were measured as change in relative resistance of the sensor. As shown in Figure 7b, it can be clearly seen that each cycle of wrist pulse waveforms had two typical peaks (P_1_ and P_2_), which were interpreted as strong peak and late systolic peak pressures, respectively. Two such key parameters, ratio and interval of the peak, are usually used for non-invasive methods of evaluating various physiological activities. According to the test results, for a healthy volunteer, by affixing sensor on the wrist, the heart rate detected was about ~70 beat/min; which is in the range of the normal heart rate for a healthy adult.

In the third case, we applied the flexible sensor for monitoring human actions such as walking, running etc. A case study of knee bending was analyzed, as shown in Figure 7c. In the study, our developed piezoresistive sensor was fixed onto a human knee, and the subject was made to perform repeated knee bending actions. The corresponding signals were observed from the resistance change in the sensor. As shown in Figure 7c, it was found that the developed sensor exhibited a good reproducibility and stability under knee flexing and bending knee actions. Last, the sensor was investigated for monitoring respiration rates. For this, the sensor was fixed on a volunteer’s chest to acquire the volunteer’s breathing rate. This was recorded as the change in the sensor’s relative resistance, which corresponded to airflow during the breathing process. The results from the four case studies provide evidence that the newly developed piezoresistive flexible sensor has broad practical applications, from monitoring human physiological activities to human machine interactions. Position of the sensors during the studies differed, and it is noteworthy to state that optimal functionality can be obtained if the sensor is fixed at the best location and direction. The poses of the sensors in the four studies are displayed with icons in Figure 7. For instance, Figure 7c, shows that the device was attached laterally onto the volunteers’ knee cap for proper data acquisition.

### 3.7. Application for the Tactile Feedback

Flexible tactile sensors, with a high sensitivity, have the capability of detecting both pressure and force, and these are increasingly desired for intelligent human–robot collaboration in robotic surgical systems. For this purpose, the flexible sensor can be adapted to perform comprehensive tasks, wherein force and pressure sensing are vital towards tactile and haptic feedback. The latter feedback is modulated from the force and pressure values exerted, and transferred to the surface of the human skin for stimuli awareness.

**Figure 8:**
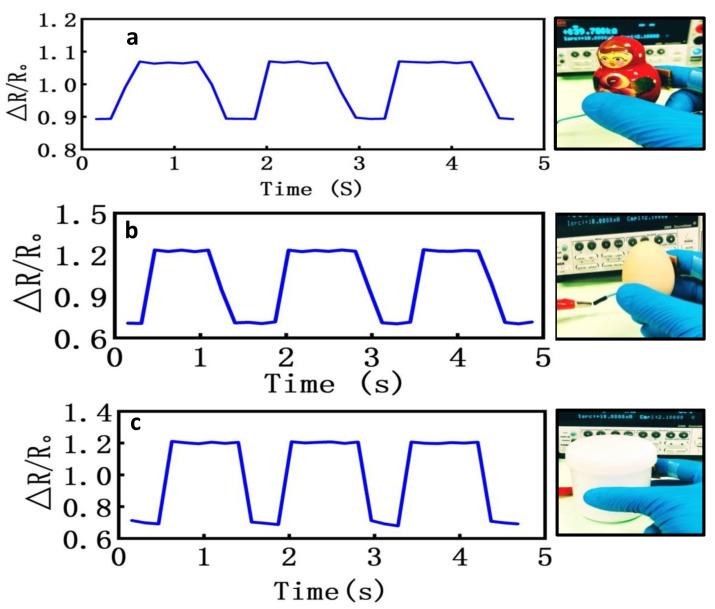
Relative resistance change monitored by the sensor attached to the fingertip when grasping a souvenir (**a**), egg (**b**), and cup (**c**) with different weights.

We also carried out experiments to demonstrate the sensing applicability of our sensor for tactile sensing and feedback. For this, we attached the developed tactile sensor at the fingertip to investigate gripping objects with different force levels exerted. In this experiment, a souvenir (29 g), egg (50 g), and cup (100 g) were used as a soft, middle, and hard objects, respectively. In this experiment, the piezoresistive tactile sensor was conformally warped around the tip of the index finger of a glove worn on a subject’s hand, and the electrical resistance change of the sensor was recorded as the subject griped each of the three objects. We first pre-determined the picking up processes normally followed to lift the objects, and carried out three repeated cycles of lifting each object. The process cycle included gripping the object, holding it, followed by releasing the object, and took a mean time of approximately 2 s per cycle. As shown in Figure 8a–c, the sensor’s response to the applied gripping force obtained similar waveforms, with different amplitude, where the amplitude implies the amount of force exerted. It can be observed that the curve of each cycle passed through the three stages.

We can explain this process as: after gripping, the force went in a stepwise manner downhill, followed by holding the load for several seconds, before again going in a stepwise manner back uphill. Compared with the process of picking up the souvenir, the sensor’s resistance variation had a larger change in the case of picking up the egg, and the largest change was observed for the process of picking up the cup. Thus, the applied force increased with increasing mass and texture (size) of the objects picked up. The possible reason for this is that when grabbing a bigger object, the surface increases the strain of the sensor’s sensitivity unit, resulting in an increased change in the sensor’s resistance. The change in resistance shows a curve that denotes the accuracies and responses to the varying forces loaded on the sensor during the picking up process, along with loading a normal pressure. Therefore, when grabbing different objects, the sensor has a small force area and a strong pressure, which results in resistance change as the subject grasped the objects. In general, the sensor’s response was almost perfectly stable and matched in the three cycles for each sensing element, with a notable variation in the resistance curves, and we were able to firmly grasp objects without causing breakage.

Finally, the experimental results illustrated that the developed piezoresistive tactile sensor holds great promise for a highly effective way of mimicking human touch, gripping, and manipulating objects during human–machine interaction, or in robot-assisted surgical systems. The sensor has can be produced at very low-cost owing to the fabrication process. Moreover, it has a fast response, high sensitivity, and excellent durability. In addition, in the near future these unique features could play a vital role for the future integration of tactile sensors for real time non-invasive intervention in robot-assisted systems.

## 4. Conclusions

In this paper, we demonstrated a facile, low-cost fabrication strategy for highly sensitive flexible piezoresistive tactile sensors, based on 3D porous PDMS sponge with a dielectric layer of CB/MWCNTs. The 3D porous structure was prepared by using a sugar sacrificial as the template. The developed sensor combines the advantages of 3D porous structure, which increases the elasticity resistance, and the hybrid synergetic effect of conductive CB/MWCNTs networks, which increase the contact area between the CB/MWCNTs particles, and reduce the distance between the inner channels of the pore’s walls under the applied pressure. Consequently, the piezoresistive tactile sensor exhibits outstanding sensing performance with excellent elasticity. Notably, it exhibits a high sensitivity of 15 kPa^−1^, a low detection limit 10 Pa, fast response time of 100 ms, and long-term durability upon 1000 cycles. The preparation strategy, and sensing mechanism, were described, and characterization of the electrical and mechanical properties of the sensor was successfully demonstrated. Furthermore, reliable performance of the proposed flexible sensor in monitoring multi-level human biological signals, such as heart rate, pulses, and respiration was successfully demonstrated. These remarkable features indicate its great potential applications in low-cost personal wearable healthcare electronics, human-machine interfacing, and robotic-assisted surgery systems.

## Figures and Tables

**Table 1:** Key performance parameters of our work and recently reported flexible piezoresistive sensors.

Materials	Sensitivity (kPa^−1^)	Response Time (ms)	Detection Limits (Pa)
3D porous MXene-sponge [53]	147 kPa^−1^–442 kPa^−1^	138 ms	9 Pa
Microstructured (MWCNT) arrays [54]	−9.95 kPa^−1^	<200 ms	—
CNT/graphene, microstructured PDMS [55]	19.8 kPa^−1^	<16.7 ms	0.6 Pa
Skin inspired [56]	35.7 kPa^−1^	107 ms	7.3 ± 1.2 Pa
Polystyrene ball rGO [57]	50.9 kPa^−1^	50 ms	3 Pa
Hydrogel-Based [58]	0.05 kPa^−1^	150 ms	—
Porous Carbon /PDMS [59]	15.63 kPa^–1^	<65 ms	—
VACNT/PDMS c composite [60]	~0.3 kPa^–1^–0.7 kPa	~162 ms	<20 Pa
Graphite/PDMS foam [61]	245 kPa^−1^	-	5 Pa
SF@MXene [62]	25.5 kPa^−1^	35–40 ms	9.8 Pa
CB/MWCNTs/PDMS (this work)	15 kPa^−1^	100 ms	10 Pa
